# Influence of pre-transplant estimated glomerular filtration rate (eGFR) on clinical outcomes after allogeneic hematopoietic cell transplantation

**DOI:** 10.1038/s41409-025-02556-8

**Published:** 2025-04-02

**Authors:** DP Nurse, H. Li, D. Cenin, S. Patel, D. Dima, J. Edwards, S. Farlow, A. Bragg, A. Mehdi, R. Hanna, SJ Rotz, D. Jagadeesh, AT Gerds, RM Dean, B. Pohlman, BK Hamilton, C. Brunstein, M. Kalaycio, CS Sauter, RM Sobecks

**Affiliations:** 1 Department of Internal Medicine, Cleveland Clinic, Cleveland, OH USA; 2 Department of Quantitative Health Sciences, Cleveland Clinic, Cleveland, OH USA; 3 Department of Hematology & Medical Oncology, Taussig Cancer Center, Blood and Marrow Transplant Program, Cleveland Clinic, Cleveland, OH USA; 4 Department of Kidney Medicine, Cleveland Clinic, Cleveland, OH USA; 5 Department of Pediatric Hematology & Medical Oncology, Taussig Cancer Center, Blood and Marrow Transplant Program, Cleveland Clinic Lerner College of Medicine of Case Western Reserve University, Cleveland, OH USA; 6 Department of Hematology & Medical Oncology, Taussig Cancer Center, Blood and Marrow Transplant Program, Cleveland Clinic Lerner College of Medicine of Case Western Reserve University, Cleveland, OH USA

**Keywords:** Stem-cell therapies, Haematological cancer

## Abstract

Pretransplant renal dysfunction has historically been associated with increased non-relapse mortality (NRM) and inferior overall survival. Novel approaches in conditioning and GVHD prophylaxis have reduced the toxicity of transplant over time, however, the impact of pre-transplant eGFR in the contemporary era is unknown. The aim of this study was to identify a pre-transplant eGFR value associated with increased transplant-related mortality. This retrospective study was performed using data from 724 adult patients who underwent first allogeneic hematopoietic cell transplant (alloHCT) from January 2012 through December 2021. The optimal pre-transplant eGFR value for risk of NRM was identified using Cox-restricted cubic spline plot analysis. Those with an eGFR <70 ml/min had the highest risk for NRM (*p* < 0.0001). Multivariate analysis confirmed that the risk of NRM remained significantly higher for eGFR <70 ml/min compared to the other higher eGFR categories, while there were no significant differences between the higher eGFR categories. Pre-transplant renal dysfunction is associated with poor outcomes after alloHCT and remains an important criterion when considering patients for transplant. Efforts to preserve renal function prior to transplant by limiting nephrotoxic exposures may have implications for optimizing outcomes after transplant, particularly in patients with other comorbidities.

## Introduction

Allogeneic hematopoietic cell transplantation (alloHCT) is a curative therapy for many hematologic diseases [1]. Although outcomes after alloHCT have improved over time [2], pre-transplant renal dysfunction remains an independent risk factor for non-relapse mortality after transplant [3]. As such, pretransplant kidney function continues to be a standard criterion for selection of patients for alloHCT [4, 5].

The Hematopoietic Cell Transplantation Comorbidity Index (HCT-CI) is a validated composite risk score based on pre-transplant comorbidities that is used to predict non-relapse mortality (NRM) in patients undergoing alloHCT [5, 6]. Within the HCT-CI, patients are considered to have renal dysfunction if they have a serum creatinine that is >2 mg/dL. More recently, estimated glomerular filtration rate (eGFR) has emerged as a better estimate of renal function in the alloHCT population, given that serum creatinine can vary widely based on age, sex, and race [3, 7–9]. Previous studies evaluating pretransplant renal dysfunction have shown that an eGFR <60 mL/min in such patients is independently associated with increased NRM and inferior overall survival (OS) [3, 7]. More recent approaches have further refined the performance of alloHCT with reduction in toxicities. In particular, the emergence of post-transplant cyclophosphamide (PTCy) as GvHD prophylaxis has enhanced outcomes [10, 11]. It is unknown whether outcomes with or without PTCy differ in relation to pre-transplant eGFR.

Despite refinements in alloHCT over time [12], we hypothesized that baseline renal function is still independently associated with differences in outcomes in the contemporary era. The aim of this study was to identify an optimal pre-transplant eGFR cut point for determining those at increased risk of HCT-related mortality. An exploratory analysis was performed to assess the effect of PTCy on outcomes in relation to pre-transplant eGFR.

## Methods

### Study design and patient population

This retrospective observational study was performed using data from adult patients who underwent first alloHCT at the Cleveland Clinic from January 2012 through December 2021. Patients ≥18 years old who underwent their first alloHCT were included. Those receiving syngeneic or umbilical cord blood grafts were excluded.

The haploidentical transplant recipients routinely received PTCy, and thus outcomes could not be compared to a group without PTCy. Therefore, a cohort of 8/8 HLA matched related and unrelated donor transplants for AML, MDS and ALL who received Busulfan (100 mg/m^2^ daily × 4 days without pharmacokinetic direction) and Fludarabine (40 mg/m^2^ daily × 4 days) conditioning was used to compare outcomes between those with and without PTCy in relation to pre-transplant eGFR.

### Study outcomes

NRM was defined as death from any cause other than disease progression or relapse. OS was estimated from the time of transplant until death from any cause. The first relapse was determined as the time of initial evidence of disease relapse after alloHCT. NRM, OS and relapse were censored at 5 years in analyses. Standard criteria were used for classifying acute and chronic GvHD [13, 14]. The first acute GVHD and first chronic GVHD after alloHCT were examined. Acute GVHD was censored at 6 months and chronic GVHD was censored at 12 months in analysis.

### Statistical analysis

Patients’ demographics and clinical characteristics before alloHCT were described descriptively using the median and interquartile range (IQR) for numerical variables and frequency. eGFR was calculated using the 2021 CKD-EPI Creatinine Equation from the Chronic Kidney Disease Epidemiology Collaboration (www.kidney.org/content/ckd-epi-creatinine-equation-2021) [15] using patients’ stable creatinine prior to hospitalization for transplant. The distribution of pre-transplant eGFR was described using median, IQR and range. The association between eGFR and risk of NRM was examined using Cox restricted cubic spline plot with competing risk of death from disease progression or relapse, and the optimal pre-transplant eGFR cut point for high-risk of NRM after alloHCT was identified at the point where both low and upper 95% CI were above 1 [16]. Patients were categorized into the following eGFR groups: <70, 70–89, 90–119, and ≥120 ml/min based upon the observed association in the spline plot along with an established cut point of 90 ml/min/1.73 m² for abnormal kidney function, and the frequency (%) of each category was reported [17]. Associations of eGFR, demographics, prognostic factors, and HCT characteristics with NRM, relapse and OS were evaluated using Cox proportional hazard model, variables with *p* < 0.05 in univariate analysis were included in multivariate survival analysis. The impact of renal function on both acute and chronic GVHD was also explored using survival analysis, and the cumulative incidence between eGFR <90 and ≥90 was compared due to the small sample size in eGFR <70 ml/min and GVHD cases. Non-transplant-related death was considered as a competing risk in the NRM analysis, and death of any cause was considered as a competing risk in both relapse analysis and GVHD analysis. A sub-analysis was performed in AML, MDS and ALL patients who received BuFlu conditioning. NRM was compared between eGFR <70 and ≥70 ml/min in patients who received PTCy and those who did not have PTCy using the Gray’s test. Due to the modest sample size, this analysis is exploratory. All analyses were performed using SAS version 9.4, two-sided *p* values are presented, *p* < 0.05 is considered statistically significant.

## Results

### Baseline characteristics

Baseline characteristics of the 724 patients included in the study are summarized in Table 1. The median (IQR) age of patients at time of transplantation was 58 (47, 65) years, with 57% males and 43% females. The median pre-transplant eGFR (IQR) was 96.7 (79.5, 107.5) ml/min, range between 14.3 to 156.9, and nearly 40% of patients had a pre-transplant eGFR <90 ml/min. A modified HCT-CI that excluded renal dysfunction was used to quantify the baseline comorbidity burden. Patients were grouped into low-risk (score of 0), intermediate-risk (score of 1-2), and high-risk (score of 3+) according to the validated index [5]. Low, intermediate, and high-risk scores corresponded with 17%, 31%, and 52% of the study population, respectively. The validated Disease Risk Index [18] was used to classify patients’ underlying disease risk, which included 17% low-risk, 56% intermediate-risk, 24% high-risk patients, and 3% the DRI was not applicable for those without hematologic malignancies. Myeloablative conditioning was used in 43% of cases and donor source included 17% haploidentical, 28% matched related, and 55% unrelated donors.

**Table 1:** Baseline patient and transplant characteristics.

Characteristics	N (%)
Total Patients	724
Male	411 (57)
Female	313(43)
Age	
18-59	400(55)
60-69	255(35)
70-79	69(10)
Race	
White	34(4.7)
Black	661(91.6)
Other	27(3.7)
Diagnosis	
AML	304(42)
MDS	167(23)
ALL	78(11)
CML	42(6)
MFB	36(5)
NHL	27(4)
AA	18(3)
Other	52(7)
Karnofsky Performance Scale	
90–100	173(24)
<90	548(76)
HCT Comorbidity Index	
Low	121(17)
Intermediate	222(31)
High	379(52)
eGFR	
<70	114 (16)
70–89	162 (22)
90–119	379 (52)
≥120	68 (9)
Disease risk index	
Low	120(17)
Intermediate	406(56)
High	174(24)
NA	24(3)
Graft Type	
Bone Marrow	264(37)
Peripheral Blood Stem Cell	460(63)
Donor Source	
Haploidentical	127(17)
Related Donor	200(28)
Unrelated Donor	397(55)
CMV donor-receipt status	
+/+	195(27)
+/−	88(12)
−/+	288(40)
−/−	153(21)
Intensity of Conditioning	
Myeloablative	312(43)
Reduced Intensity	412(57)
Conditioning Regimen	
Busulfan/Cyclophosphamide	180
Busulfan/Cyclophosphamide/ATG	2
Busulfan/Cyclophosphamide/Thio/ATG	1
Busulfan/Fludarabine	256
Busulfan/Fludarabine/ATG	40
Busulfan/Fludarabine/Thio	1
Cyclophosphamide/ATG	5
Cyclophosphamide/TBI	2
Cyclophosphamide/TBI/ATG	11
Cyclophosphamide/TBI/ECP	3
Fludarabine/ATG	1
Fludarabine/Cyclophosphamide/TBI	79
Fludarabine/Cyclophosphamide/Thio/TBI/ATG	1
Fludarabine/Melphalan/Alemtuzumab	3
Fludarabine/TBI	85
Fludarabine/TBI/Alemtuzumab	1
Etoposide/TBI	52
GVHD Prophylaxis	
Cyclosporine/MMF	125
Cyclosporine/Bortezomib/MMF	1
Cyclosporine/Methotrexate	5
Cyclosporine/Tacrolimus/Methotrexate	218
Cyclosporine/Methotrexate/ECP	3
Tacrolimus/MMF	163
Tacrolimus/Methotrexate	150
Tacrolimus/Methotrexate/Bortezomib	6
Tacrolimus/Mini-Methotrexate/MMF	45
Tacrolimus/Methotrexate/Maraviroc	4
Sirolimus/Cyclosporine/MMF	3

*MMF* mycophenolate mofetil, *ECP* extracorporeal photopheresis, *ATG* antithymocyte globulin, *Thio* thiotepa, *TBI* total body irradiation.

### Effect of pre-transplantation renal dysfunction on non-relapse mortality and overall survival

**Fig. 1:**
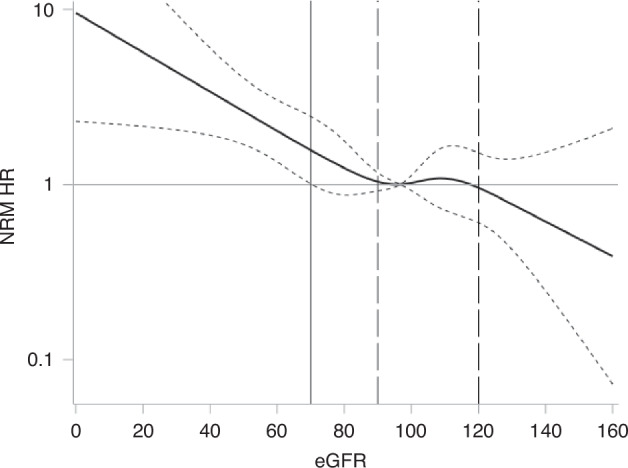
Cox restricted cubical spline plot with risk of NRM by eGFR level. The Cox-restricted cubical spline plot shows that the risk of NRM was significantly higher with an eGRF below 70. The left vertical line shows the eGFR 70 ml/min cutoff, while the bold horizontal down sloping line and the dotted lines above and below show the hazard ratio and both low and upper 95% confidence intervals are above 1.

Association between pre-transplant eGFR level and NRM is illustrated in Fig. 1. The Cox restricted cubical spline plot shows that the risk of NRM increased at eGFR <90 ml/min, and the risk became significantly higher with an eGFR <70 ml/min (HR and 95% CI above 1.57, 1.01–2.45), and reduced risk was observed with an eGFR ≥120, although not statistically significant. Based on the observed association, patients were grouped into eGFR <70, 70-89, 90-119 and ≥120 ml/min which represented a distribution of 15.8% (*N* = 114), 22.4% (*N* = 162), 52.4% (*N* = 379) and 9.4% (*N* = 68) of the study population, respectively.

**Fig. 2:**
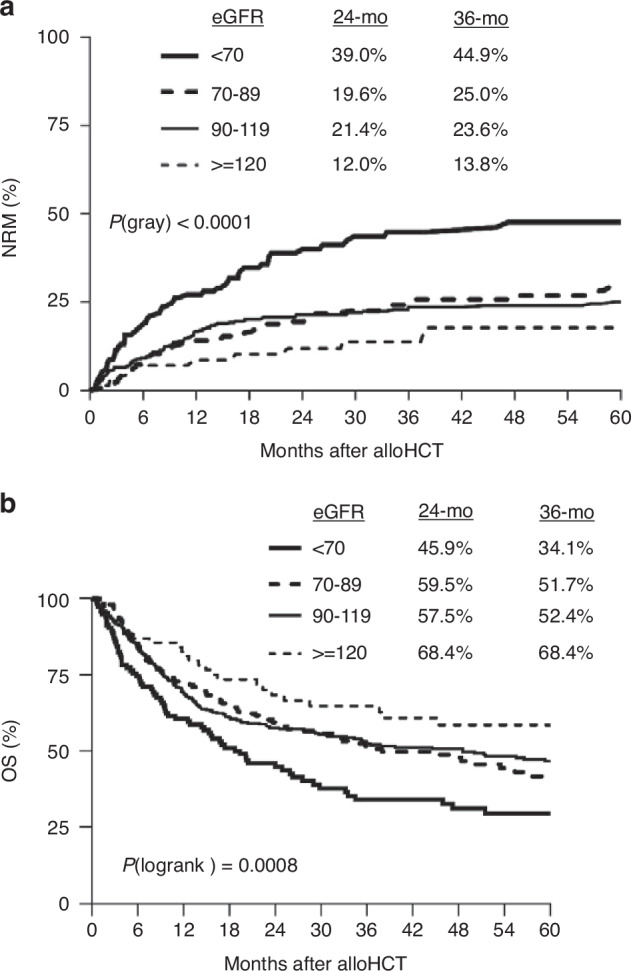
Cumulative incidence of NRM and overall survival by eGFR level. **a** Cumulative incidence of NRM by eGFR level. Patients with eGFR <70 ml/min had the highest NRM cumulative incidence among eGFR categories. **b** Cumulative incidence of overall survival by eGFR level. Patients with eGFR <70 had the worst OS among eGFR categories.

Figure 2a shows that the cumulative incidence of NRM was significantly different among eGFR groups (*p* < 0.0001). Patients with eGFR <70 ml/min had the highest NRM cumulative incidence among eGFR categories. The estimated 24-month cumulative incidence was 39.0% in the eGFR <70 ml/min category compared to 19.6% in the 70–89, 21.4% in the 90–119 and 12.0% in the ≥120 ml/min cohorts. The risk of NRM in the eGFR <70 ml/min group was significantly higher than the other higher eGFR categories, while differences among the higher eGFR categories were not statistically significant.

Univariate and multivariate analyses for associations of eGFR and other factors with NRM are presented in Table 2. The results remained the same after adjusting for age, Karnofsky performance status (KPS), intensity of conditioning, graft type, donor source, busulfan/fludarabine (BuFlu) conditioning and HCT-CI without renal function [Adjusted HR (95% CI) compared to the higher eGFR categories: 1.7 (1.1–2.6) to eGFR 70-89, 1.9 (1.3–2.7) to eGFR 90–119, and 2.7 (1.3–5.7) to eGFR ≥120]. There were no significant differences in NRM among the other higher pre-transplant eGFR groups.

**Table 2:** Factors associated with NRM.

	Univariate		Multivariate	
Characteristics	HR (95% CI)	*P* value	Adjusted HR (95% CI)	*P* value
eGFR				
≥120	1		1	
90–119	1.58 (0.85, 2.95)	0.15	1.45 (0.72, 2.91)	0.30
70-89	1.71 (0.89, 3.3)	0.11	1.59 (0.77, 3.26)	0.21
<70	3.41 (1.79, 6.51)	0.0002	2.72 (1.3, 5.67)	0.008
<70 vs 70–89	1.99 (1.33, 2.98)	0.0008	1.71 (1.14, 2.59)	0.01
<70 vs 90–119	2.16 (1.52, 3.05)	<0.0001	1.88 (1.30, 2.71)	0.0008
70–89 vs 90–119	1.08 (0.75, 1.55)	0.68	1.10 (0.76, 1.59)	0.62
Age category				
18–59	1		1	
60–69	1.23 (0.91, 1.68)	0.18	1.00 (0.67, 1.52)	0.98
70–79	1.88 (1.21, 2.9)	0.005	1.46 (0.85, 2.53)	0.17
Sex, Male	1.01 (0.76, 1.35)	0.92		
Race				
Black	1			
Caucasian	1.33 (0.62, 2.85)	0.46		
Other	1.56 (0.57, 4.24)	0.38		
KPS ≥ 90 vs <90	0.54 (0.4, 0.73)	<0.0001	1.64 (1.21, 2.23)	0.001
Intensity of conditioning, RIC vs MAC	1.48 (1.10, 2.00)	0.01	0.87 (0.53, 1.42)	0.57
Graft type, PSC vs BM	2.20 (1.57, 3.09)	<0.0001	2.28 (1.47, 3.52)	0.0002
Donor source				
Haplo	1		1	
RD	1.55 (0.94, 2.55)	0.08	2.18 (1.24, 3.83)	0.007
URD	1.83 (1.16, 2.87)	0.009	2.43 (1.42, 4.15)	0.001
Bu/Flu use	1.46 (1.1, 1.94)	0.009	0.65 (0.41, 1.04)	0.07
PTCy use	0.77 (0.55, 1.07)	0.11		
HCT-CI (without renal)				
Low	1		1	
Intermediate	1.25 (0.77, 2.02)	0.363	1.18 (0.72, 1.92)	0.51
High	1.65 (1.06, 2.56)	0.026	1.43 (0.91, 2.24)	0.12
CMV donor–receipt				
+/+	1			
+/−	0.91 (0.53, 1.57)	0.74		
−/+	0.97 (0.68, 1.37)	0.85		
−/−	0.95 (0.63, 1.42)	0.79		
DRI				
High	1			
Intermediate	1.05 (0.74, 1.5)	0.79		
Low	1.11 (0.71, 1.73)	0.66		
NA	1.03 (0.43, 2.5)	0.94		

In multivariate analysis, risk of NRM in eGFR<70 remained significantly higher compared to other categories after adjusting for age, KPS, intensity of conditioning, graft type, donor source, Busulfan/Fludarabine use and HCT-CI without renal function. Differences among other categories were not significant.

Similar patterns were observed in the OS analysis. OS was also significantly different among eGFR groups (Fig. 2b, *p* = 0.0008). The estimated 24-month OS was 45.9% in the eGFR <70 ml/min category compared to 59.5% in the 70–89 ml/min, 57.5% in the 90–119 ml/min and 68.4% in the ≥120 ml/min cohorts.

In both univariate and multivariate analyses (Table 3), OS for patients with an eGFR <70 was significantly worse than all three higher eGFR categories. Early death in the eGFR <70 ml/min cohort was nearly 3 times higher compared to those with an eGFR ≥120, and nearly 2 times higher compared to those with eGFRs of 70–89 and 90–119 ml/min after adjusting for age, KPS, intensity of conditioning, graft type, busulfan/fludarabine use, HCT-CI without renal function, and DRI that potentially associated with OS in univariate analysis.

**Table 3:** Factors associated with overall survival.

	Univariate		Multivariate	
Characteristics	HR (95% CI)	*P* value	HR (95% CI)	*P* value
eGFR				
≥120	1		1	
90–119	1.46 (0.97,2.20)	0.072	1.26 (0.81,1.95)	0.31
70–89	1.52 (0.98,2.35)	0.064	1.28 (0.79,2.08)	0.31
<70	2.28 (1.45,3.56)	<0.001	1.94 (1.17,3.21)	0.01
<70 vs 70–89	1.50 (1.10, 2.06)	0.01	1.51 (1.09, 2.1)	0.01
<70 vs 90–119	1.56 (1.19, 2.05)	0.001	1.54 (1.15, 2.07)	0.004
70–89 vs 90–119	1.04 (0.8, 1.35)	0.77	1.02 (0.78, 1.34)	0.89
Age (years)				
18–59	1		1	
60–60	1.33 (1.07,1.65)	0.012	1.21 (0.88,1.65)	0.24
70–79	1.60 (1.13,2.25)	0.008	1.33 (0.87,2.05)	0.19
Gender				
Female	1			
Male	1.04 (0.84,1.28)	0.73		
Race				
Black	1			
Caucasian	0.99 (0.61,1.59)	0.96		
Other	0.79 (0.37,1.68)	0.54		
KPS				
90–100	1		1	
<90	1.59 (1.27,1.99)	<0.001	1.53 (1.20,1.93)	<0.001
Conditioning Intensity				
MAC	1		1	
RIC	1.40 (1.13,1.73)	0.002	0.93 (0.63,1.36)	0.71
Graft type				
BM	1		1	
PBSC	1.43 (1.15,1.78)	0.001	1.14 (0.84,1.56)	0.40
Donor source				
Haploidentical	1		1	
Related donor	0.97 (0.71,1.33)	0.84	0.97 (0.71,1.33)	0.84
Unrelated donor	1.03 (0.77,1.36)	0.85	1.03 (0.77,1.36)	0.85
Conditioning regimen				
Bu/Flu	1		1	
Other	1.27 (1.04,1.57)	0.022	0.91 (0.67,1.24)	0.56
PTCy for GvHD prophylaxis				
No	1			
Yes	1.07 (0.85,1.34)	0.57		
HCT-CI without renal				
Low	1		1	
Intermediate	1.06 (0.76,1.48)	0.75	1.04 (0.74,1.47)	0.83
High	1.50 (1.10,2.03)	0.009	1.387 (1.02,1.90)	0.04
Donor-Receipt CMV status				
−/−	1			
+/+	1.22 (0.91,1.65)	0.19		
+/−	1.10 (0.73,1.66)	0.64		
−/+	1.09 (0.83,1.45)	0.53		
DRI				
High	1.60 (1.16,2.20)	0.004	1.45 (1.03,2.04)	0.033
Intermediate	0.99 (0.73,1.32)	0.92	0.88 (0.65,1.21)	0.44
Low	--		--	
NA	0.48 (0.21,1.12)	0.089	0.53 (0.20,1.42)	0.21
Age at HCT, per 10 years	1.18 (1.09,1.28)	<0.001		

In multivariate analysis, risk of OS in eGFR<70 also remained significantly higher compared to other categories after adjusting for age, KPS, intensity of conditioning, graft type, and Bu/Flu use, HCT-CI without renal function and DRI. Differences among other categories were not significant.

Pre-transplant eGFR was not significantly associated with relapse (data not shown). It also was not associated with acute GVHD and chronic GVHD, and the cumulative incidences were similar between eGFR <90 and ≥90 ml/min (data not shown). The most common causes of death within 5 years after alloHCT were relapse (52.9%), infection (17.0%) and organ failure (7.7%). Relapse was less common (38.4% vs 56.5%) while the infection was more common (21.9% vs 15.8%) in the eGFR<70 ml/min cohort compared to the ≥70 ml/min group, while organ failure was similar (8.2% vs 7.5%).

### Effect of using PTCy-based GVHD prophylaxis on alloHCT outcomes in patients with underlying renal dysfunction

**Fig. 3:**
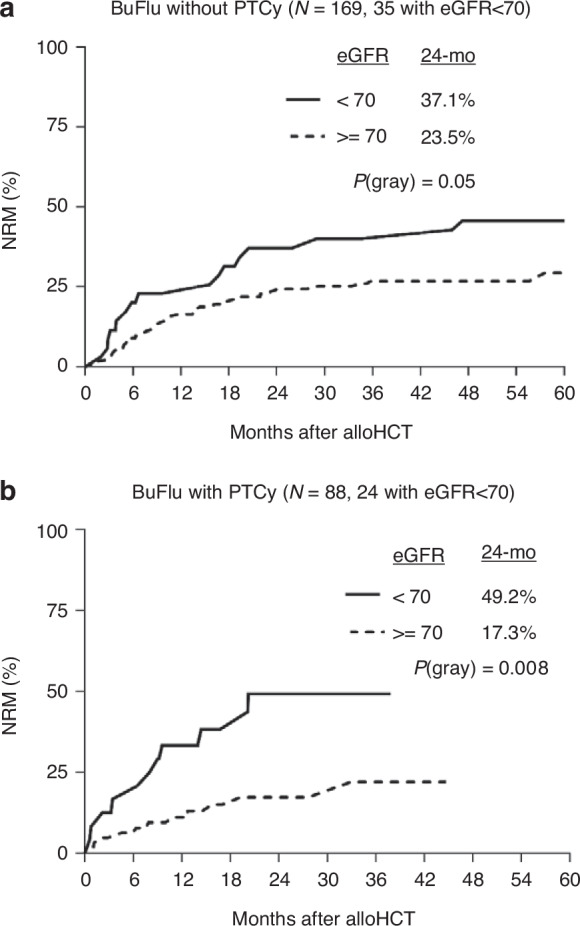
NRM in AML, MDS, and ALL patients with Bu/Flu conditioning with or without PTCy. **a** NRM in AML, MDS, and ALL patients with Bu/Flu conditioning without PTCy. **b** NRM in AML, MDS, and ALL patients with Bu/Flu conditioning with PTCy. The difference in NRM between those with a baseline eGFR <70 or ≥70 ml/min was greater in the PTCy cohort than for those who did not receive PTCy.

An exploratory analysis was performed to compare NRM between those with a baseline eGFR <70 and ≥70 ml/min in relation to the use of PTCy. This analysis included 257 patients with AML, MDS, and ALL who underwent matched related or unrelated alloHCT from 2019 to 2021 with BuFlu conditioning. Among the 169 who received BuFlu without PTCy, 35 had an eGFR <70 ml/min. There were 88 patients who received BuFlu with PTCy, of which 24 had an eGFR <70 ml/min. No differences in NRM were observed when comparing PTCy vs. no PTCy for those with an eGFR <70 ml/min. Similarly, when considering those with an eGFR >70 ml/min there were no differences in NRM between the PTCy and no PTCy cohorts. When considering those who received PTCy, the difference in NRM between eGFR <70 and ≥70 ml/min was 49.2% vs. 17.3% at 24 months (*p* = 0.008) while for those who did not receive PTCy this was 37.1% vs 23.5%, respectively (*p* = 0.05), as shown in Fig. 3.

## Discussion

The current study found that a pre-transplant eGFR <70 ml/min was the optimal eGFR cut point for predicting high risk of HCT-related mortality. However, relapse was not associated with pre-existing renal dysfunction.

Assessment of pre-existing kidney function is an important criterion when considering patients for transplant. The Hematopoietic Cell Transplantation Comorbidity Index (HCT-CI) is a tool used to determine the risk of NRM after transplant based on pre-existing comorbidities. Within the HCT-CI, renal dysfunction is defined as a serum creatinine >2, history of a kidney transplant, or the need for dialysis [5]. A serum creatinine >2 is a very limited definition of kidney dysfunction, as serum creatinine can vary widely in individuals depending on several factors, including race, sex, age, and muscle mass. Baseline eGFR, rather than creatinine, has been shown to be a better indicator of pre-transplant renal function in the alloHCT population [3, 7].

Assessment of kidney function prior to transplant should include a careful assessment of GFR. Creatinine-based estimating equations are the most readily available means of assessing GFR and remain the initial step. In select patients where non-GFR creatinine determinants may be important to consider (e.g., extremes of either increased or decreased muscle mass), cystatin C-based equations need to be utilized for a more accurate GFR estimation. Importantly, the gold standard for GFR assessment remains iothalamate or iohexol clearance-based measurements. In select patients, particularly those where the estimated GFR may fall close to the cutoff values, iothalamate/iohexol-based GFR assessment might be needed. This may allow for better patient selection and counseling prior to alloHCT.

An eGFR <60 ml/min is commonly defined in clinical practice as moderate kidney dysfunction, and is the threshold for stage 3 chronic kidney disease [17]. In the general population, an eGFR <60 ml/min has been shown to be an independent predictor of mortality risk [19]. In previous studies evaluating the effect of renal dysfunction on outcomes after alloHCT, a pretransplant eGFR <60 ml/min was used to categorize patients with moderate kidney dysfunction [3, 7, 20, 21]. Using data from an alloHCT population and not from the general population alone, our study found that a pre-transplant eGFR <70 ml/min (using the most contemporary 2021 CKD-EPI Creatinine Equation, which excludes race) was the optimal cut point for identifying those at increased risk of HCT-related mortality.

Our study corroborates prior evidence that pre-existing renal dysfunction as measured by eGFR is a prognostic factor for mortality after alloHCT. A retrospective, registry study by Farhardfar et al. with over 13,000 patients from an older transplant time interval found that a pre-transplant eGFR <60 ml/min was associated with increased risk of NRM and post-transplant renal failure requiring dialysis [3]. Shouval et al. reported more than 1200 cases in which an eGFR <60 ml/min was also associated with increased NRM and overall mortality [7]. In contrast, some smaller studies found that an eGFR <60 ml/min was not associated with worse NRM and OS in the reduced intensity conditioning setting with fludarabine/melphalan or 200 cGy total body irradiation with or without fludarabine [20, 21]. While these results may be valuable for select populations, they are not likely applicable to a broader population and different conditioning regimens.

To our knowledge, our series is the first study to compare outcomes between those who were treated with and without PTCy in relation to pre-transplant eGFR. Our analysis observed that in the setting of matched related or unrelated donor alloHCT with BuFlu conditioning, a pre-transplant eGFR <70 ml/min had significantly worse NRM with or without PTCy. As compared to those who did not receive PTCy, the PTCy group had a greater difference in NRM between those with an eGFR <70 and ≥70 ml/min. This may be related to better tolerance of PTCy in those with higher baseline eGFRs, which may be associated with less GvHD-related mortality. Although our results suggest there is no mortality detriment to using PTCy-based GVHD prophylaxis in patients with reduced renal function, assessments of larger populations are necessary to better define the impact in disease-specific groups.

It is important to note that although these patients received PTCy as part of their regimen for GVHD prophylaxis, most of these patients also received tacrolimus and mycophenolate mofetil (MMF). Given the renal toxicity of calcineurin inhibitors (CNIs), exposure to tacrolimus may have precipitated further renal dysfunction in these patients [22, 23]. In attempts to avoid the negative effects of CNIs, there have been multiple studies that have evaluated CNI-free GVHD prophylaxis [24–29]. One recent phase III trial published by Luznik et al. evaluated outcomes of 327 alloHCT patients who received single-agent cyclophosphamide or a T-cell-depleted peripheral blood stem cell graft as compared to tacrolimus + methotrexate for GvHD prophylaxis and found that PTCy alone was associated with more acute GVHD. However, the rates of chronic GvHD, relapse-free survival, and overall survival were similar. The authors concluded that the single-agent PTCy regimen could potentially be offered to patients with contraindications to CNIs [24]. CD3^+^TCRαβ/CD19^+^ depletion has also been an effective alternative approach to avoid CNIs with HLA mismatched family or unrelated donor alloHCT [30, 31]. Evidence is lacking regarding CNI-free GVHD prophylaxis in patients with underlying renal dysfunction, and this presents an important opportunity for future research. Currently, if such patients are otherwise felt to be appropriate candidates for alloHCT then the use of a reduced-intensity conditioning approach with limiting nephrotoxic agents and CNI-free GVHD prophylaxis may be most appropriate.

This retrospective, single-center study had some limitations. Data on the underlying cause and duration of many patients’ pre-transplant renal dysfunction was not known. The sample size for some of the disease groups included in the analysis may have limited the generalizability of the outcome results for these populations. The exploratory analysis of patients receiving PTCy-based GVHD prophylaxis was limited to only AML, MDS and ALL patients who underwent HLA-matched related and unrelated donor transplants with busulfan and fludarabine (BuFlu) conditioning. The finding of no difference in NRM between those treated with or without PTCy may not be generalizable for other disease groups, HLA mismatched grafts, reduced intensity conditioning, other myeloablative conditioning regimens or older patients.

In conclusion, our study found that an eGFR <70 ml/min is an independent prognostic factor for mortality after alloHCT, which may have important implications when selecting patients for transplant. Future investigation with larger subgroup analyses regarding specific diagnoses, conditioning regimens, and transplant modalities may further identify which patients are more appropriate for transplant and those who may benefit from alternative treatment approaches. Our results also suggest that additional efforts to preserve renal function prior to transplant by limiting nephrotoxic exposures may have implications for optimizing outcomes after transplant, particularly in patients with other comorbidities.

## Data Availability

All data were obtained from the Cleveland Clinic’s Unified Transplant Database.
